# Thoracic endovascular repair of a rare case of leaking aortic arch intramural hematoma secondary to Giant cell arteritis

**DOI:** 10.1186/s42155-019-0052-6

**Published:** 2019-02-21

**Authors:** Abhijit Salaskar, Farzad Najam, Elizabeth Pocock, Shawn Sarin

**Affiliations:** 1 Interventional Radiology Department, George Washington University Hospital, Washington, DC USA; 2 Cardiothoracic Surgery Department, George Washington University Hospital, Washington, DC USA

**Keywords:** Giant cell arteritis, GCA, Thoracic aortic aneurysms, Aortic aneurysms, Intramural hematoma, Aortitis, Endovascular aortic repair, TEVAR

## Abstract

**Background:**

Traditionally thoracic aortic aneurysms (TAA) secondary to Giant Cell Arteritis (GCA) were treated with resection and open repair. However no prior studies have reported an aortic intramural hematoma (IMH) as a presentation of GCA or outcome of thoracic endovascular aortic repair (TEVAR) in TAA or IMH secondary to GCA.

**Case presentation:**

A 59 year old female, nonsmoker, non-hypertensive, non-diabetic with a known history of GCA, temporal arteritis on prednisone presented with shortness of breath & chest pain. Chest CT revealed aortic arch IMH and large left hemothorax. CTA confirmed distal aortic arch focal dilation, a focal intimal irregularity in the distal aortic arch and extensive IMH without any active extravasation or signs of aortitis. Patient underwent an urgent TEVAR without oversizing the aortic landing zones. Post TEVAR aortogram showed exclusion of the site of IMH origin and dilated aortic arch segment by the stent and absence of active extravasation. One month post-TEVAR CTA showed patent stent graft with resolution of IMH and hemothorax. One year after TEVAR, patient remained asymptomatic.

**Conclusion:**

GCA can present as an IMH secondary to underlying chronic vasculitis. When endovascular repair is considered, great care should be taken not to grossly oversize aortic landing zones.

## Background

Aortic involvement has been estimated to occur in up to 18% of patients with Giant cell arteritis (GCA). Patients with GCA are 17.3 times more likely to develop thoracic aortic aneurysm (TAA) compared to the general population of the same age and sex (Evans et al, 1995). (1) Traditionally TAA secondary to GCA have been treated with resection and open aortic repair. However an intramural hematoma (IMH) as a presentation of GCA has not been reported. Also no prior studies have addressed the outcome of thoracic endovascular aortic repair (TEVAR) in TAA or IMH secondary to GCA. This case is of a patient who had a rare presentation of focal distal transverse aortic arch dilation and contained leaking IMH secondary to GCA which was successfully treated by TEVAR.

## Case presentation

**Fig. 1:**
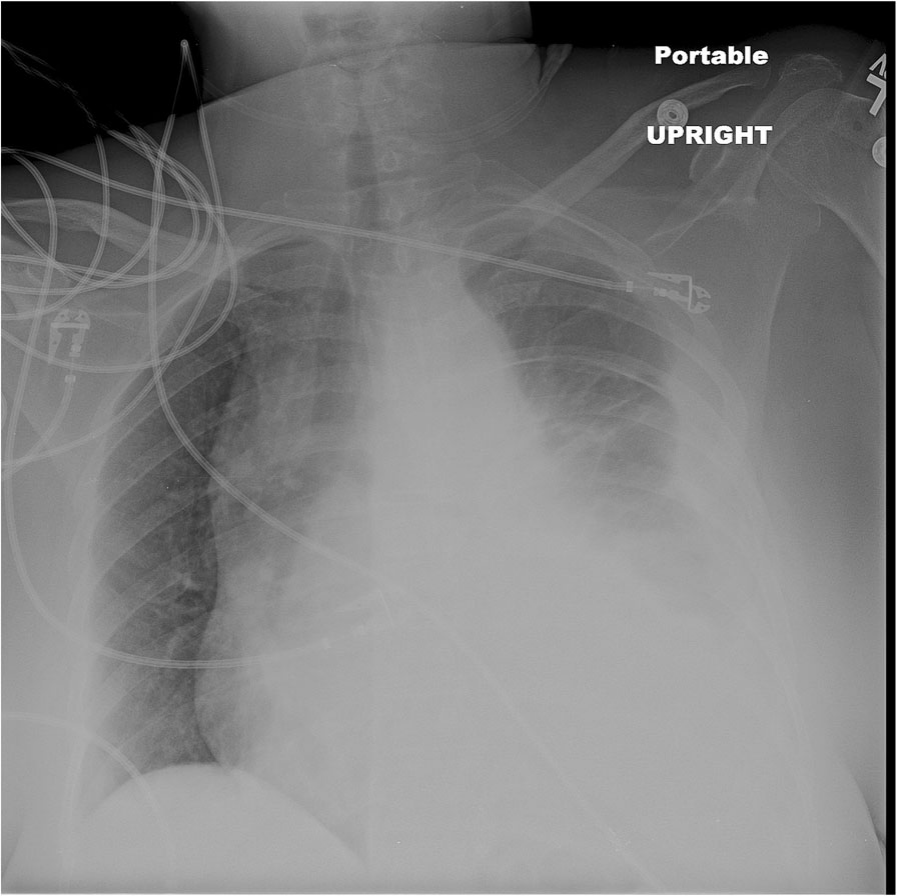
Initial chest x ray showing widened mediastinum, opacification of left hemithorax & rightward mediastinal shift

**Fig. 2:**
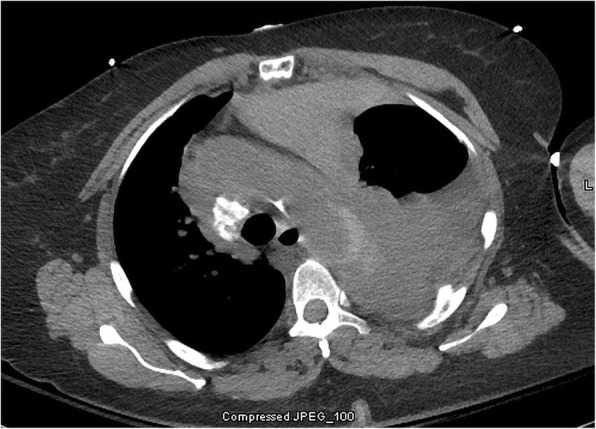
Pre-TEVAR CTA (axial view of non-contrast phase) showing crescentic aortic IMH originating in the distal aortic arch near the takeoff of the left subclavian artery and large left sided hemothorax

**Fig. 3:**
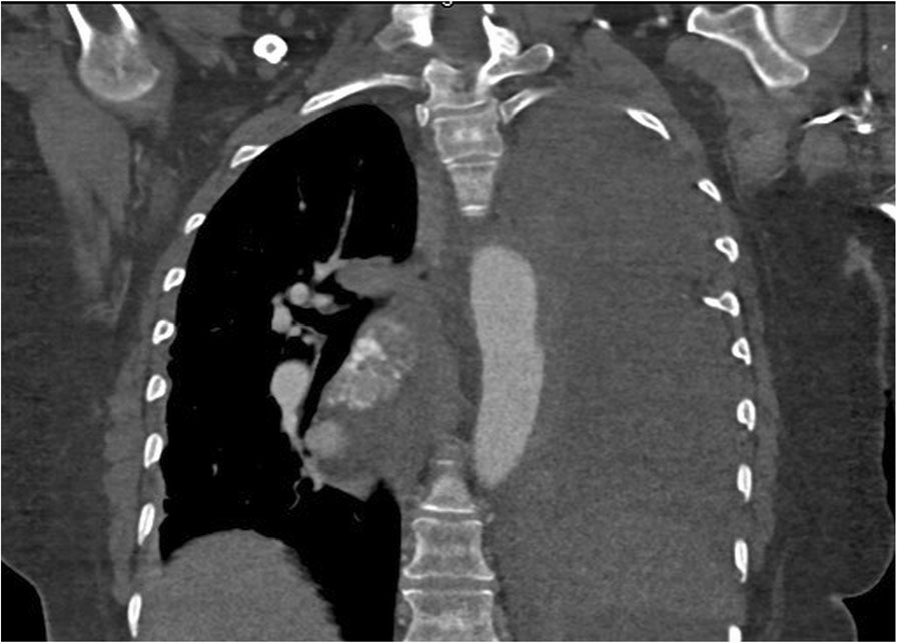
Pre-TEVAR CTA (coronal view) showing focal intimal irregularity in the distal aortic arch. No evidence of active extravasation

**Fig. 4:**
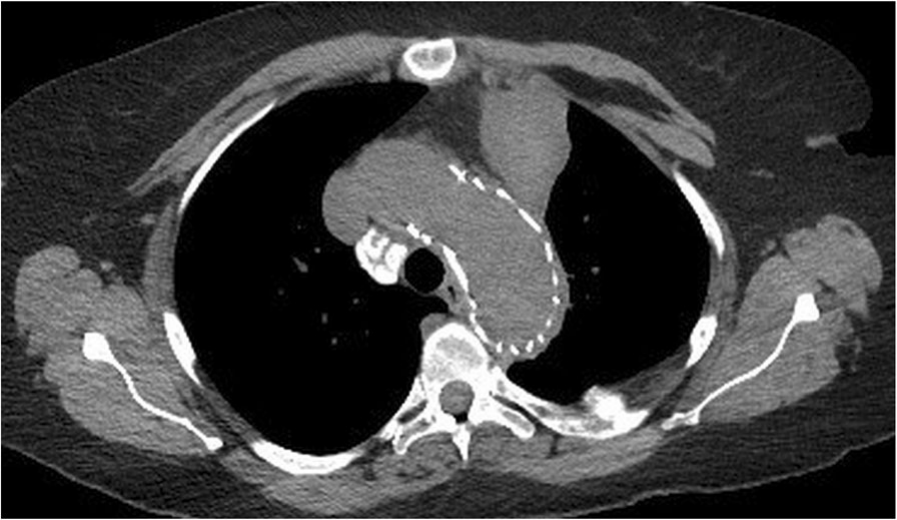
Follow up CTA (axial view) one month after TEVAR showing an aortic stent graft and resolution of intramural hematoma

**Fig. 5:**
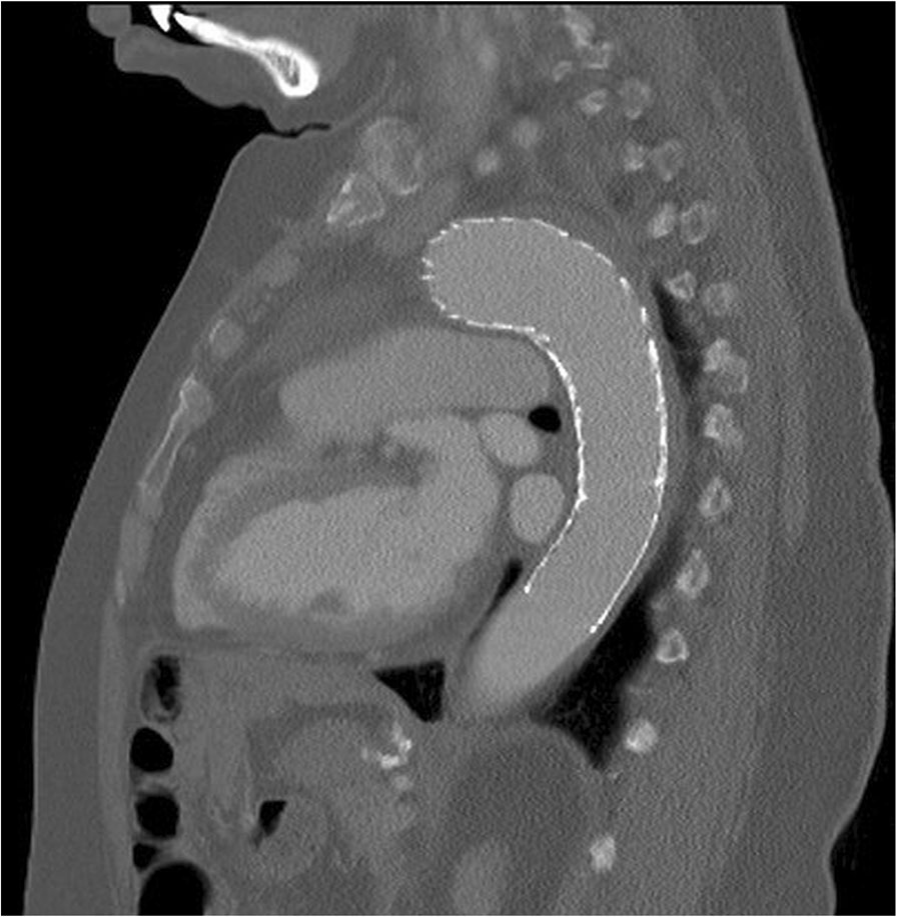
Follow up CTA (sagittal view) one month after TEVAR showing widely patent aortic stent graft without any evidence of extravasation or endoleak

A 59 year old female who is nonsmoker, non-hypertensive, non-diabetic with a known history of Giant Cell Arteritis (GCA), temporal arteritis on prednisone for 6 years and systemic lupus erythematous, presented with sudden onset shortness of breath, left sided chest pain and back pain. Initial chest x ray revealed widened mediastinum, opacification of left hemithorax & rightward mediastinal shift (Fig. 1). CT chest revealed crescentic aortic IMH and large left sided hemothorax (Fig. 2). Subsequent aortic dissection protocol CTA confirmed a focal dilation of distal transverse aortic arch (maximum diameter of 3.7 cm), a focal intimal irregularity in the distal transverse aortic arch and leaking IMH extending from the origin of the left subclavian artery to the origin of celiac artery without any evidence of active extravasation (Fig. 3). There was no CT evidence of active aortitis or atherosclerosis. The patient was hemodynamically stable. Patient was urgently taken to an interventional radiology suite with an intention to perform an endovascular repair of this contained aortic rupture. Through a right groin access, an aortogram was performed and it showed a patent three vessel aortic arch, a distal transverse aortic arch dilatation near the origin of left subclavian artery and absence of active extravasation. It was suspected that this focally dilated segment of distal transverse aortic arch near the origin of left subclavian artery was responsible for large IMH and left sided hemothorax. Hence prior to aortic stent graft insertion, cerebral angiography was performed. It confirmed that the right and left vertebral arteries were communicating at the basilar confluence. The right groin access was then serially dilated to accommodate a 24 French sheath. Then two overlapping Gore thoracic stent grafts 37–37 mm × 15 cm and 31–26 mm × 10 cm were deployed within aortic arch intentionally covering the left subclavian artery and descending thoracic aorta respectively. We intentionally did not oversize the aortic stent grafts to reduce the risk of aortic intimal injury and possible dissection. Post stenting aortogram showed complete exclusion of dilated aortic arch segment. Follow up aortogram, initially showed intermittent active extravasation which ceased once the aortic stent graft was balloon molded. Then left hemothorax was decompressed by placement of a large bore chest tube. On postoperative day 1, repeat CTA showed widely patent aortic graft, significant decrease in the left hemothorax and resolution of mediastinal shift (Fig. 4). Follow up CTA after one month showed widely patent aortic stent graft and complete resolution of aortic intramural hematoma (Fig. 5). There was no evidence of active extravasation or endoleak. One year after the TEVAR, patient remained asymptomatic. Patient never reported any arm claudication.

## Conclusion

The frequency of involvement of thoracic aorta in Giant cell arteritis has not been accurately reported in the available literature, however it has been considered relatively low. The detection of early involvement of the thoracic aorta by vasculitis is difficult to discern clinically because the aortic inflammation does not produce recognizable symptoms and biopsy of the aorta is not feasible. Based on the available literature, median time from diagnosis of GCA to diagnosis of thoracic and abdominal aortic aneurysm were 10.9 years and 6.3 years, respectively (Marie et al., 2009). No such data is available about IMH secondary to GCA. Our patient had a rare presentation of focal distal transverse aortic arch dilation and an extensive IMH. Patient did not have atherosclerotic disease. As per the literature, fluorodeoxyglucose positron emission tomography can detect aortitis in early stages, however we did not have any CT evidence of aortitis (Borchers & Gershwin, 2012).

Patients presenting with symptomatic large vessel GCA with or without involvement of cranial vessel GCA are typically treated with corticosteroids at the same doses as patients with cranial GCA. Patients with symptomatic large vessel GCA may benefit from higher corticosteroid dose and it may prevent further complications such as aortic dissections or rupture, however it has not been studied or proven in the available literature. The long term evolutions of large artery disease secondary to GCA and its treatment outcomes have been very sparsely reported.

In one prior study, out of 34 patients who had aortitis detected on a routine CT scan at the time of diagnosis of GCA, 9% patients had resolution of aortitis, 47% patients had improvement in aortitis, and 41% patients had stable aortitis at 6 months follow up. Four out of these 34 patients developed of TAA at 16 months of follow up (Nuenninghoff et al., 2003). This and other series have reported that, atleast 40% of patients with TAAs required surgery for TAA resection and/or aortic valve replacement or repair (Borchers & Gershwin, 2012; Evans et al., 1994; Marie et al., 2009) to prevent development of complications such as of aortic dissection or rupture which increase the mortality (Nuenninghoff et al., 2003). Similarly urgent management in warranted in case of IMH to prevent aortic dissection or rupture.

In the latest review discussing the management strategies of 156 histologically proven cases of aortitis, type 3 disease i.e. thoraco-abdominal aortic disease was treated by bypassing and stenting of greater vessels, before carrying out thoracoabdominal aortic repairs to the greater vessels or concurrent prosthetic material bypasses to visceral arteries or left subclavian bypass (Svensson et al., 2015). In our case, we deployed aortic stent graft intentionally covering left subclavian artery after confirming the patent communication of the right and left vertebral arteries at the basilar confluence. This allowed us to effectively exclude the area of focal dilation and intimal irregularity in the distal transverse aortic arch. Even in the absence of CT evidence of active aortitis such as in our case the aorta is expected to be fragile with long standing GCA, hence great care should be taken not to grossly oversize aortic landing zones. TEVAR can be an effective treatment of diseases causing chronic vasculitis such as GCA to prevent continuous destruction of aortic wall and to treat the complications such as rupture, obviating the need for highly morbid open repair and bypass procedures. Thus GCA can present as an intimal injury and intramural hematoma secondary to underlying chronic vasculitis. When endovascular repair is considered, great care should be taken not to grossly oversize aortic landing zones.

## Abbreviations

GCA: Giant cell arteritis
IMH: Intramural hematoma
TAA: Thoracic aortic aneurysms
TEVAR: Thoracic endovascular aortic repair
