# Correction to: Thoracic endovascular repair of a rare case of leaking aortic arch intramural hematoma secondary to Giant cell arteritis

**DOI:** 10.1186/s42155-020-0102-0

**Published:** 2020-02-24

**Authors:** Abhijit Salaskar, Farzad Najam, Elizabeth Pocock, Shawn Sarin

**Affiliations:** 1 Department of Interventional Radiology, George Washington University Hospital, Washington, DC USA; 2 Department of Cardiothoracic Surgery, George Washington University Hospital, Washington, DC USA

**Correction to: CVIR Endovasc (2019) 2:9**

**https://doi.org/10.1186/s42155-019-0052-6**

‘In the published article (Salaskar et al. 2019) the statement under the subheading ‘Consent for publication’ is incorrect:

“IRB approval not needed for this type of submission at our institution.”

should read:

“Informed consent for publication of this case report and its accompanying images has been obtained from the patient”
